# Editorial: Antibody-based novel target immunotherapy in hematological malignancy

**DOI:** 10.3389/fonc.2025.1727734

**Published:** 2025-11-13

**Authors:** Weijie Cao, Yingmei Li, Haizhou Xing, Zhongxing Jiang, Jifeng Yu

**Affiliations:** Department of Hematology, The First Affiliated Hospital of Zhengzhou University, Zhengzhou, China

**Keywords:** antibody-based therapy, hematological malignancy, immunomodulation, tumor microenvironment, resistance mechanism, combination immunotherapy, CD58, DLBCL

Antibody-based immunotherapy remains a cornerstone of modern treatment for B-cell and other hematologic malignancies, yet the discipline is evolving rapidly beyond single-agent anti-CD20 or PD-1 antibodies toward rational combination regimens, biomarker-guided precision, and strategies designed to overcome or preempt resistance. Unlike conventional chemotherapy, monoclonal antibodies and their derivatives enable targeted elimination of malignant clones while simultaneously recruiting the host immune system (1). However, their efficacy is often constrained by mechanisms of acquired resistance, immune escape, and the complex, dynamic architecture of the tumor microenvironment (TME) (2).

This Research Topic seeks to deepen our understanding of antibody-driven therapies for hematologic cancers and to refine their clinical application. The four featured papers—comprising a case report of lenalidomide plus PD-1 blockade in relapsed/refractory classical Hodgkin lymphoma (cHL), a complex case of DLBCL with sequential antibody-based regimens and targeted maintenance, a bioinformatics analysis revealing hypoxia-induced mechanisms of rituximab resistance in DLBCL, and a focused review highlighting CD58 as a prognostic and immunotherapy-relevant antigen—collectively delineate the trajectory and future direction of antibody therapeutics. The following summary integrates their key insights and translational relevance.

## Clinical innovation: combination strategies to re-sensitize the immune response

Dong et al. described a heavily pretreated, elderly patient with relapsed/refractory cHL who achieved durable clinical benefit from lenalidomide combined with PD-1 inhibition after multiple prior therapies, including brentuximab vedotin and PD-1 monotherapy. The case underscores two practical points: first, immunomodulatory drugs (IMiDs) such as lenalidomide can enhance innate and adaptive immune function—particularly NK and T-cell activity—thereby strengthening antibody-dependent cytotoxicity and checkpoint efficacy; second, carefully chosen, well-tolerated combinations can achieve meaningful disease control for patients ineligible for intensive salvage therapy. Although anecdotal, this observation provides a clinical rationale for combining IMiDs with checkpoint inhibitors, particularly in the inflammatory microenvironment characteristic of cHL.

## Real-world complexity: antibody use in multimodal therapy

The DLBCL case involving multiple primary tumors illustrates the enduring centrality of antibody-based regimens in multidisciplinary care (4). Rituximab-containing combinations produced sustained remission, while relapse was successfully managed through alternative antibody-based chemotherapies and subsequent maintenance with a BTK inhibitor. This treatment sequence—initial antibody therapy, salvage with antibody-containing regimens ± targeted agents, followed by maintenance—exemplifies the personalized therapeutic sequencing that defines modern hematologic oncology, while also highlighting both the durability and limitations of antibody-based interventions amid clonal evolution and comorbid malignancies.

## Mechanisms of resistance: hypoxia-driven remodeling of CD20 signaling

In their bioinformatics study, Yao et al. revealed that hypoxia within the tumor microenvironment induces a transcriptional signature associated with rituximab resistance in DLBCL. The authors identified a “DLBCL–hypoxia overlap” (DHO) gene set—including *LGALS1*, *TIMP1*, *ANXA1*, *STAP1*, *GPNMB*, and *CDCA7*—that interacts with BCR and PI3K–AKT signaling pathways, both known to attenuate antibody-mediated cytotoxicity. These findings suggest that integrating anti-CD20 antibodies with inhibitors of hypoxia-adaptive signaling or metabolic modulators may restore therapeutic sensitivity and that DHO-related biomarkers could help identify patients at higher risk for treatment failure.

## CD58: a key mediator and predictive biomarker in antibody-based cellular therapy

Cao et al. provided an in-depth review of CD58 (LFA-3), an adhesion molecule critical for forming immunologic synapses through CD2 interactions. CD58 facilitates T- and NK-cell activation and is thus essential for the activity of bispecific antibodies and CAR-based therapies. Loss or mutation of CD58 correlates with poor prognosis and resistance to CAR-T and bispecific T-cell engager (BiTE) therapy—often mediated by increased PD-L1 expression and immune escape. Importantly, CD58 suppression can occur via EZH2-associated epigenetic silencing, suggesting that expression may be pharmacologically restored. Consequently, CD58 serves as both a biomarker for therapeutic selection and a potential target for intervention.

## Integrative perspective

Together, these studies outline a unified framework for advancing antibody-based immunotherapy in hematologic malignancies:

1. Biomarker-guided personalization: Integration of hypoxia-related signatures with CD58 profiling can inform optimal therapeutic choices—anti-CD20 for hypoxia-low/CD58-intact disease, combination or alternate pathways for hypoxia-high or CD58-suppressed cases.
2. Rational combinations: The lenalidomide plus PD-1 case demonstrates the clinical viability of pairing IMiDs with antibodies or checkpoint inhibitors. Future trials should incorporate biomarkers such as CD58 and hypoxia gene signatures as stratification variables.
3. Targeting hypoxia-mediated pathways: Dual inhibition of PI3K/AKT signaling alongside anti-CD20 therapy may overcome hypoxia-driven resistance.
4. Restoring immune synapse integrity: Routine CD58 assessment should guide the use of bispecific or CAR-based therapies, while epigenetic priming may counteract CD58 loss.

## Conclusion

Antibody therapeutics in hematologic malignancies have entered an era of integration—where monoclonal antibodies, immunomodulators, and small molecules converge on shared immune mechanisms. The collective contributions of Dong et al., Zhang et al., Yao et al., and Cao et al. demonstrate the necessity of combining clinical innovation, mechanistic discovery, and biomarker-driven design. Despite ongoing challenges, particularly in acute myeloid leukemia (AML), continued exploration of novel targets and rational combinations is poised to enhance the efficacy of bispecific antibodies (BsAbs) and other antibody-based modalities in relapsed/refractory disease. Future research should prioritize biomarker validation and combination trials integrating checkpoint blockade, metabolic inhibitors, and IMiDs to optimize response and overcome resistance (3, 4).
